# Retinal Gatekeepers: Molecular Mechanism and Therapeutic Role of Cysteine and Selenocysteine

**DOI:** 10.3390/biom15081203

**Published:** 2025-08-21

**Authors:** Eleonora Maceroni, Annamaria Cimini, Massimiliano Quintiliani, Michele d’Angelo, Vanessa Castelli

**Affiliations:** Department of Life, Health and Environmental Sciences, University of L’Aquila, 67100 L’Aquila, Italy; eleonora.maceroni@guest.univaq.it (E.M.); annamaria.cimini@univaq.it (A.C.); massimiliano.quintiliani@univaq.it (M.Q.); michele.dangelo@univaq.it (M.d.)

**Keywords:** cysteine, selenocysteine, oxidative stress, retina, retinal diseases

## Abstract

Oxidative stress is a key contributor to retinal degeneration, as the retina is highly metabolically active and exposed to constant light stimulation. This review explores the crucial roles of cysteine and selenocysteine in redox homeostasis and retinal protection. Cysteine, primarily synthesized via the transsulfuration pathway, is the rate-limiting precursor for glutathione (GSH), the most abundant intracellular antioxidant. Selenocysteine enables the enzymatic activity of selenoproteins, particularly glutathione peroxidases (GPXs), which counteract reactive oxygen species (ROS). Experimental evidence from retinal models confirms that depletion of cysteine or selenocysteine results in impaired antioxidant defense and photoreceptor death. Furthermore, dysregulation of these amino acids contributes to the pathogenesis of age-related macular degeneration (AMD), retinitis pigmentosa (RP), and diabetic retinopathy (DR). Therapeutic approaches including N-acetylcysteine, selenium compounds, and gene therapy targeting thioredoxin systems have demonstrated protective effects in preclinical studies. Targeting cysteine and selenocysteine-dependent systems, as well as modulating the KEAP1–NRF2 pathway, may offer promising strategies for managing retinal neurodegeneration. Advancing our understanding of redox mechanisms and their role in retinal cell viability could unlock new precision treatment strategies for retinal diseases.

## 1. Introduction

**Figure 1:**
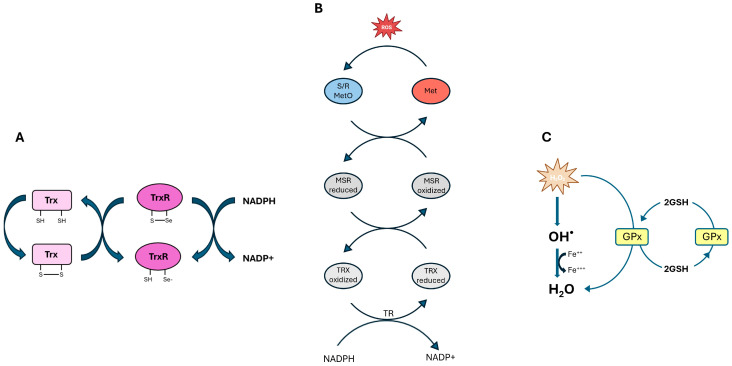
(**A**). The mechanism of action of the TRX redox system. (**B**). A schematic illustration of the redox cycle of MSR and MSRB in reducing MetO. (**C**). An illustration of GPX, which protects cells by converting hydrogen peroxide into water through the Fenton reaction. “Fe^++^” and ”Fe^+++^” respectively represent “Fe^2+^” and “Fe^3+^”, as well as the Fenton reaction involved.

Oxygen is a fundamental element for life. Its role is essential not only in aerobic respiration but also in various biosynthetic processes such as detoxification, defense mechanisms, and metabolic activities crucial for maintaining the physiological state of cells. However, oxygen also plays a crucial role in the aging process and the development of oxidative stress-related diseases by forming reactive oxygen species (ROS) [1]. ROS include superoxide anions, hydrogen peroxide, and hydroxyl radicals. These species, primarily generated by normal mitochondrial activity, have important roles in cellular signaling and immune function when present at low to moderate levels [2]. An imbalance between ROS production and antioxidant defenses leads to oxidative stress. When ROS levels exceed the cell’s ability to neutralize them, oxidative damage can occur, affecting lipids, proteins, and DNA, thereby increasing the risk of mutations and cell death [3]. The eye is susceptible to oxidative damage due to its high metabolic activity and constant exposure to light radiation. Persistent oxidative exposure can lead to cumulative damage affecting multiple ocular structures, from the eye surface to the retina [4]. Antioxidant enzymes such as superoxide dismutase, catalase, and various oxidoreductases, including selenoproteins, play a pivotal role in maintaining ROS at steady-state levels. It has been shown that the enzymatic activity of selenoproteins is largely attributed to the presence of selenocysteine (Sec) at their active sites, enabling the detoxification of ROS and cellular protection [5] (Figure 1).

Given the impact of oxidative stress on the functionality of retinal pigment epithelium (RPE) cells, several lines of research have focused on identifying oxidative mechanisms to better understand retinal degeneration processes and explore potential protective treatments. Depletion of glutathione (GSH) and cysteine has been shown to correlate with RPE cell damage and death [6].

Based on this background, this review aims to explore how cysteine and selenocysteine contribute to the protection and regulation of retinal function, as well as the role of oxidative stress in the development of retinal diseases.

## 2. The Retinal Gatekeepers

In mammals, the pathway that allows cysteine biosynthesis is the transsulfuration pathway. This process involves cystathionine, which allows for the transfer of sulfur from homocysteine. From dietary methionine, homocysteine is obtained, which, once transformed into cystathionine by cystathionine β-synthetase (CBS), is activated by cystathionine γ-lyase (CSE) to produce cysteine [7]. Cysteine plays a key role in redox regulation as it is crucial for glutathione availability (GSH). It is known that 50% of the cysteine derived from the transsulfuration pathway is destined for GSH synthesis in the liver [8]. This importance is highlighted by the strong link between abnormal cysteine metabolism, and consequently GSH, and disruptions in the redox balance [9]. Since cysteine is involved in a wide range of processes that regulate the physiological state of the cell, the transsulfuration pathway is subject to tight internal control. Both CBS and CSE undergo various post-translational modifications, which lead to changes in their enzymatic activity and cellular localization. It is particularly interesting to understand the specific conditions that favor cysteine production through CSE, rather than the synthesis of H_2_S, GSH, or taurine [10]. The CBS enzyme contains a heme group that plays an important regulatory role. Its enzymatic activity is modulated through binding with specific endogenous ligands. CBS preferentially catalyzes the reaction between homocysteine and serine to produce cystathionine, as serine has a higher affinity than cysteine. Similarly, CSE has a greater affinity for cystathionine and thus catalyzes its cleavage to produce cysteine. In the presence of high levels of nitric oxide or carbon monoxide, the binding of these ligands to the heme group inhibits CBS activity, leading to the accumulation of homocysteine and promoting the production of H_2_S by CSE [11]. CSE is highly inducible in response to environmental and intracellular stresses, which vary depending on the cell type and tissue. At the genetic level, the expression of CSE is primarily regulated by SP1 (Specificity Protein 1), which maintains its basal expression [12].

GSH is the most abundant antioxidant in mammalian cells and, in addition to its detoxifying role, it is also important in several metabolic reactions [13,14]. GSH is primarily synthesized by glutamate cysteine ligase (GCL), which catalyzes the reaction between glutamate and cysteine to form γ-glutamylcysteine. To obtain GSH, the enzyme glutathione synthetase adds glycine. The amount of available cysteine is a limiting factor for GSH synthesis, so it is not surprising that import/export of cysteine are regulated processes for maintaining GSH/GSSG levels [13]. Within cells, reduced glutathione can react with oxidizing agents either spontaneously or through reactions catalyzed by glutathione peroxidase (GPX) [15]. This process leads to the formation of glutathione disulfide, consisting of two GSH molecules joined by a disulfide bridge. GSSG can be actively exported out of the cell or interact with sulfhydryl groups present in proteins (PSH), giving rise to mixed disulfide (PSSG) and contributing to the depletion of intracellular GSH [16]. GSSG can be converted back to GSH through the action of the enzyme glutathione reductase (GR), which uses NADPH as a cofactor [17]. To prevent oxidative damage to proteins, GSH also intervenes in transsulfuration reactions, catalyzed GLRX, by protecting protein thiol groups [18]. Furthermore, glutathione S-transferase (GST) can conjugate GSH to numerous hydrophobic and electrophilic molecules, such as drugs, carcinogens, and products of oxidative metabolism, facilitating their detoxification and elimination from the cell [16].

The preservation of redox homeostasis also depends critically on the regulation of the selenoproteome. Unlike the canonical genetic code described in the 1960s, selenocysteine is incorporated at the UGA codon, which ordinarily functions as a stop codon in most mRNAs [19]. In humans, there are twenty-five genes defined as selenoprotein genes, in which UGA codons are recoded to specify selenocysteine [20]. For the insertion of selenocysteine, the presence of the SECIS element is essential; it is in the 3′-UTR of all selenoprotein mRNAs [21]. The presence of the SECIS element alone determines the recoding of UGA [22]. The SECIS element is essential for the translation of selenoproteins. It plays a key role in the recruitment of specific factors, including the SECIS binding protein (SBP2) and Sec-specific translation elongation factor (eEFSec), which collaborate in the insertion of selenocysteine. As mentioned, without a functional SECIS, UGA would be interpreted as a translation stop signal, preventing the proper synthesis of selenoproteins. Therefore, the element SECIS is crucial in the synthesis of selenoproteins and, therefore, for the performance of their biological function [23].

**Figure 2:**
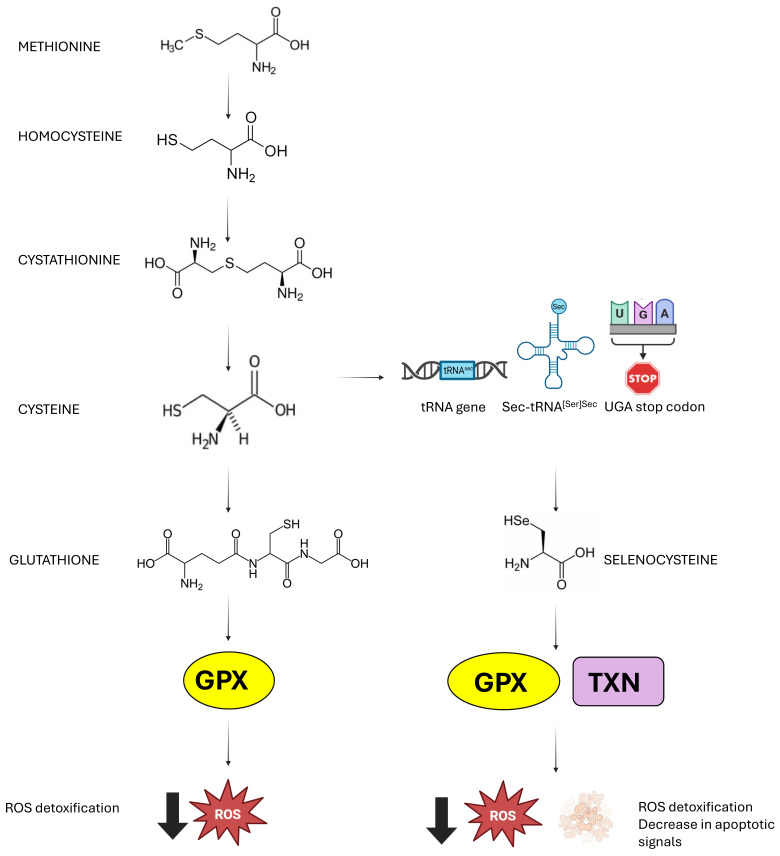
Biosynthesis of cysteine and selenocysteine. The downward arrow indicates a decrease.

In addition to the SECIS sequence, the Sec-tRNA[Ser]Sec plays a primary role and is characterized by unique features compared to other tRNAs [24,25]. This tRNA gene is present as a single copy in the genome and is sufficient for all twenty-five selenoproteins. The human Sec-tRNA[Ser]Sec is encoded by the TRU-TCA1-1 gene located on chromosome 19, and there is a related pseudogene found on chromosome 22. Various regulatory mechanisms regulate selenoprotein expression [26]. The antioxidant role attributed to the selenoproteome is due to the presence of selenoenzymes: five GPX, three thioredoxin reductases (TrxR), and methionine sulphoxide reductase 2 (MsrB) [27,28]. GPX enzymes reduce peroxide and hydroperoxides of organic or phospholipid nature using reduced glutathione [29]. Specifically, GPX1 represents the main defense system among selenoproteins against situations of significant oxidative stress [30]. While MsrB plays a specific role in the reduction in methionine sulphoxide, Trx enzymes act on multiple targets, including oxidized thioredoxins, hydrogen peroxide, and phospholipid peroxides [31,32]. Selenoprotein P also shows a marginal antioxidant role, even though it is primarily responsible for the uptake of plasma selenium [33] (Figure 2).

In the table below (Table 1), the main human selenoproteins involved in redox regulation in the retina are summarized, highlighting their enzymatic functions, cellular localization, and antioxidant roles.

The thioredoxin (TXN) and glutaredoxin (GLRX) systems are two evolutionarily conserved antioxidant mechanisms that play a crucial role in protecting cells from oxidative stress and in regulating redox-sensitive cellular processes. In the TXN system, an important role is played by thioredoxin reductase (TXNRD), which contains selenocysteine and reduces oxidized thioredoxin using NADPH. The GLRX system utilizes NADPH to reduce oxidized glutathione to GSH through glutathione reductase, and the reduced GSH, in turn, acts on glutaredoxin.

Once TXN and GRLX are reduced, they can act by reducing disulfide bonds in specific proteins, ensuring their active and thus protective form [43]. The first study to investigate the thioredoxin system in the retina was conducted by Hansson and Holmgren, who analyzed the modulation of the expression of insulin-like growth factor 1 (IGF1) during retinal development in rats [40]. IGF1 is a known substrate of TXN, which led the authors to examine the distribution of TXN, TXNRD, and ribonucleotide reductase (RNR). Immunoreactivity showed that TXN and TXNRD are evenly expressed by various retinal cell populations during development, including retinal ganglion cells (RGCs), cells of the inner nuclear layer (INL), and photoreceptors. However, with retinal maturation, a marked reduction in TXN and TXNRD expression is observed in photoreceptors, suggesting a stage-specific role during development. In parallel, ribonucleotide reductase (RNR), which requires TXN as an electron donor for DNA synthesis, was detected in the early postnatal days (PND) in the INL and photoreceptors, but progressively disappears by PND12, remaining expressed only in some scattered retinal endothelial cells. This finding is consistent with previous observations indicating colocalization between TXN and RNR only in actively proliferating tissues, but not in the mature retina. These pieces of evidence suggest that, in the post-developmental retina, TXN may serve functions beyond the reduction in RNR, including modulation of redox status, cellular signaling, and antioxidant protection, functions that align with cell cycle arrest and terminal differentiation of retinal precursors during maturation [40,41]. By analyzing retinal damage caused by ischemia–reperfusion conditions, several biomarkers of oxidative stress have been identified [44]. Ohira and collaborators proposed direct involvement of the TXN system in retinal protection. Their studies showed that the expression of TXN increased in the RPE following ischemic episodes and prolonged light exposure. Furthermore, the research group examined the effect of a prostaglandin E1 (PGE1) analog, a compound known for its applications in the treatment of vascular diseases, including ischemic retinopathy [45]. In a murine model of ischemia–reperfusion injury, treatment with the PGE1 analog maintained stable TXN levels for up to 14 days, whereas in untreated mice, TXN levels progressively declined [46]. These observations suggest that TXN may contribute to the neuroprotective effects of PGE1, mitigating ROS-induced damage and preserving retinal tissue integrity. Experiments conducted in murine models via intravitreal injection of recombinant TXN1 or transgenic overexpression of TXN1 showed significant preservation of photoreceptors, suggesting a strong protective effect [47,48]. However, this effect was completely absent when using a mutated form of TXN1 in which the cysteines of the active site (Cys32 and Cys35) were replaced by serines (C32S/C35S mutation). This finding confirms that the presence of these two cysteines is essential for TXN’s antioxidant function and its ability to protect photoreceptors from oxidative damage. In another study, Cao and collaborators observed impaired expressions of TXN1 and TXNRD1 in the tubby mouse model. Treatment with sulforaphane, an activator of the erythroid nuclear factor 2-related factor 2 (NRF2) antioxidant pathway, restored TXN1 and TXNRD1 levels at both the transcriptional and protein levels, indicating that this redox axis can be therapeutically modulated [42]. Subsequently, the same group generated transgenic mice in which TXN1 expression was controlled by the β-actin promoter, resulting in widespread retinal distribution (inner segment, OPL, IPL, and GCL). In these animals, TXN1 expression conferred both structural and functional protection of photoreceptors. At the molecular level, this protection was associated with increased expression of the neurotrophic factors BDNF and GDNF, activation of pro-survival pathways (AKT, RAS, RAF1, ERK), and inhibition of the apoptotic ASK1/JNK signaling pathway [49]. These findings support the hypothesis that TXN1 acts not only as an antioxidant but also as a modulator of neuroprotective factor transcription, with potential therapeutic application in degenerative retinopathies. In the retina of rd1 mice, a model of retinal degeneration, a reduction in total GPX activity was observed, accompanied by increased lipid peroxidation, indicating impaired antioxidant capacity [50]. Among the isoforms of the GPX family, GPX4 is the most studied in humans and has three splicing variants, localized in the cytoplasm, mitochondria, and nucleus [38]. In retinal tissue, GPX4 is widely expressed, particularly in the inner segments of photoreceptors, but is also found in the RPE and choroid. Specific removal of GPX4 in photoreceptors led to lipid peroxide accumulation and cell death already in the early stages of retinal development, demonstrating that GPX4 is indispensable for photoreceptor maturation and survival [39]. Furthermore, functional studies have shown that overexpression of GPX1 or GPX4 in RPE cells confers marked resistance to oxidative stress.

These observations confirm that GPX4 plays a key role in retinal antioxidant defense, acting as a guardian against ferroptosis and other forms of photoreceptor degeneration.

As already mentioned, ROS are not only harmful but also play crucial roles in cell signaling under both physiological and pathological conditions. This occurs mainly through covalent modification of cysteine residues in proteins. The thiol (-SH) groups of cysteines are highly reactive and easily oxidized by ROS. These modifications are mostly reversible, allowing cysteine to function as a redox switch. However, when oxidation progresses to the formation of sulfonic acid (–SO_3_H), the modification becomes irreversible, resulting in a definitive loss of protein function. These mechanisms finely regulate various cellular processes, including proliferation, differentiation, stress response, and cell death, assigning cysteine a critical role in intracellular redox signaling. In the retinal context, TXN donates electrons to peroxiredoxins (PRDX), enzymes that convert hydrogen peroxide into water, contributing to the maintenance of redox balance. A key example is Apoptosis Signal-Regulating Kinase 1 (ASK1), a kinase of the MAP3K (mitogen-activated protein kinase) family, involved in activating the JNK and p38 signaling pathways responsible for ER stress response and apoptosis.

Under physiological conditions, TXN1 interacts with ASK1 by forming a disulfide bond between the cysteines of its active site and cysteine C250 in the N-terminal region of ASK1, inhibiting its kinase activity [51,52]. This mechanism is particularly relevant in various animal models of ocular diseases, such as glaucoma and ischemic retinal damage, where dissociation of the TXN–ASK1 complex promotes neuronal death. Moreover, TXN plays a central role in reducing protein S-nitrosylation, a modification typical of inflammatory states that can impair protein function. In a murine model of retinal degeneration induced by N-methyl-N-nitrosourea (MNU), increased S-nitrosylation was observed following inflammation and light exposure, but this could be partially counteracted by TXN’s ability to remove such modifications and restore protein function [53,54,55]. The role of glutaredoxin (GLRX) in the retina is more controversial, but some studies suggest significant involvement under pathological conditions.

Mieyal’s group demonstrated that GLRX1 activity increases in the diabetic retina and Müller cells cultured under high-glucose conditions. In this context, overexpression of GLRX1 is associated with enhanced activation of the transcription factor NF-κB, known for its proinflammatory functions. Moreover, Müller cells overexpressing GLRX1 release high amounts of interleukin-6 (IL-6), triggering an autocrine and paracrine feedback loop that amplifies NF-κB activity and GLRX1 expression itself. This effect appears to be mediated by GLRX’s ability to remove GSH residues from IκB kinase (IKK), the main repressor of NF-κB, thereby promoting its activation [56].

## 3. Retina and Oxidative Stress: A Delicate Balance

The retina is a tissue that is characterized by a high metabolic demand. To maintain its proper function, the retina activates numerous processes, resulting in a glucose consumption that surpasses that of the brain [57]. This high energy demand results from the continuous processing of visual stimuli and the maintenance of membrane potential by the photoreceptors. These processes require considerable ATP consumption and result in the physiological production of ROS. Although they are normally neutralized by antioxidant systems, excessive amounts of ROS can overcome cellular defenses and cause damage. During the visual cycle, photoreceptors transform light stimuli into electrical impulses. At the same time, visual pigments in the RPE undergo conformational changes in response to light. A significant example is lipofuscin, a chromophore presents in the RPE, which, upon absorption of high-energy photons, especially blue light, acts as a primary photo-oxidant [58]. Even in the absence of light, large quantities of ATP are utilized; in fact, photoreceptors in the dark must maintain an active membrane potential through ion pumps. Large consumption of ATP is associated with maintaining the ionic homeostasis necessary for the proper functioning of neurotransmitters [59]. A physiological consequence of these processes is the generation of ROS, which, as mentioned, do not cause damage as long as they are maintained at physiological levels [60]. The eye has a fine defense system against oxidative stress, and it is interesting to note that these systems differ depending on the region being considered [61,62].

At the retinal level, several oxidative stress processes have been identified using models of light-induced retinal degeneration. Studies reported that in such cases, aberrant ROS generation cannot be compensated by antioxidant defenses and eventually can lead to photoreceptor death. ROS normally affects DNA, proteins, and lipids. Their interaction with DNA can result in irregular gene expression or cell death, while interaction with lipids and proteins leads to abnormalities in signal transduction, protein structure, enzyme activity, especially at the mitochondrial level, and effects on the cytoskeleton and cell organelles [63,64,65,66,67]. The results of these processes are reflected in the establishment of inflammatory conditions, autophagy, and endoplasmic reticulum stress, as well as the triggering of apoptosis when the damage is so heavy that compensation is not possible. Another serious consequence is the breakdown of mitochondria and the interruption of cell–cell interactions. In addition, there is a tissue-organ disorganization risk [68,69].

The outer portions of the phagocytosed photoreceptors contain high concentrations of polyunsaturated fatty acids and represent the main endogenous source of ROS in the RPE. Furthermore, the RPE is exposed to high oxygen tension due to its proximity to the capillaries of the choroid [70].

People chronically exposed to video terminal equipment have been reported to experience eye problems, including dryness, fatigue, and blurred vision [71]. Photoreceptors are particularly sensitive to excessive light exposure, and it is known that alterations in the RPE increase the probability of retinal diseases [72]. In 2014, Liu and co-workers developed an in vitro model of light damage to photoreceptors by exposing the ARPE-19 cell line to light insults. It was observed that inhibition of proliferation and the establishment of apoptotic mechanisms occurred in a directly proportional manner to exposure time and light intensity. In addition, elevated VEGF levels in these cells were recorded due to the induced damage. Among the reported effects were an increase in intracellular ROS levels and the percentage of senescent cells [73]. Another study has shown that exposure to visible light causes oxidative damage in RPE cells by promoting the formation of advanced glycation end-products and simultaneously reducing levels of free GSH [74,75]. Another study investigated the effect of different types of oxidative stress using primary RPE culture. Specifically, cells were exposed to high concentrations of oxygen, hydrogen peroxide, and paraquat. By ELISA assays, protein carbonyl contents were assessed to quantify oxidative damage. This study concluded that different antioxidants affect different cellular districts, suggesting that endogenous antioxidant defenses are more effective for mitochondria and endoplasmic reticulum damage but less against cell surface damage [76].

At the level of the RPE, the most present and crucial antioxidant is GSH. It is well established that this compound exerts a protective effect when administered under oxidative stress conditions, and that its depletion is closely associated with cell death [77,78]. Lack of GSH can lead to cell death in the RPE through various processes: apoptosis, autophagy, ferroptosis, and necrosis [79,80].

Physiologically, iron, mainly present as Fe^3+^ and Fe^2+^, is transported in the blood bound to transferrin, a glycoprotein that binds to Fe^3+^ and delivers it to cells via the transferrin receptor. After absorption, iron is released and reduced to Fe^2+^ to enter the cytoplasm, where it can be used or stored in storage proteins such as ferritin, thus preventing excess free iron that can cause oxidative stress and ferroptosis, an iron-dependent form of cell death [81].

A 2018 paper concluded that GSH depletion may correlate with ferroptosis, autophagy, and stress-induced premature senescence (SIPS) in RPE cells. The proposed mechanism suggests that GSH serves as the substrate for GPX4 in counteracting the accumulation of lipid ROS. In addition to promoting oxidative stress, this study links ROS depletion to the induction of ferroptosis in RPE cells. Specifically, the use of cysteine-free medium or drug blockade of de novo GSH synthesis led to cell death. Treatment with ferroptosis inhibitors (Ferrostatin-1 and Liproxstatin-1) protected the cells from this outcome. Interestingly, DFO, an iron chelator, allowed cells to survive in the absence of cysteine. Probably due to the accumulation of a small percentage of iron in the cytosol and organelles, DFO appears to localize primarily in lysosomes, where it protects cells by chelating iron. Moreover, the use of autophagy inhibitors also promoted cell survival, highlighting the important role of this process. It can be concluded that GSH depletion results in cell death by depleting the lysosomal iron reserve [70].

It is well known that mitochondria are key players in apoptosis/necrosis processes and that they are also involved in the production of ROS in the RPE. In a Sun and colleagues’ study, cell death induced by oxidative stress was found to result from lipid peroxidation rather than the accumulation of ROS at the mitochondrial level. Considering the crucial importance of GSH in cellular redox balance and the recognized link between oxidative stress and autophagic processes, it can be hypothesized that the oxidative imbalance caused by GSH reduction represents a central node in the interplay between ferroptosis and autophagy [70].

In protecting photoreceptors, a crucial role is played by N-acetyl-L-cysteine (NAC). N-acetyl-L-cysteine (NAC), a modified form of the amino acid L-cysteine, serves as a therapeutic agent in medicine. It is primarily utilized to break down mucus and to manage cases of drug poisoning or overdose. NAC can be administered through oral ingestion, intravenous injection, or inhalation [82]. In 2017, Nakamura et al. developed a mouse model of retinal damage induced by blue LED light. Specifically, animals were exposed to light at intensities of 400 or 800 lux for two hours. After five days, the electroretinogram (ERG) and the measurement of the thickness of the outer nuclear layer of the retina (ONL) made it possible to assess the light-induced damage. In addition, decreased expression of S-opsin and abnormal localization of rhodopsin in the ONL were found. Intraperitoneal administration of NAC protected the retina from induced damage in a dose-dependent manner. Specifically, the treatment preserved both the expression of S-opsin and the correct localization of rhodopsin [83].

Lutein/zeaxanthin also counteracts oxidative stress and preserves light-exposed photoreceptors. Since it has been found that a similar effect can be achieved by blocking the angiotensin II type 1 receptor (AT1R), it can be concluded that oxidative stress and inflammation may contribute to the same pathological mechanism, acting on common biological pathways [66]. This theory is supported by the observation that, under less acute exposure conditions, photoreceptor apoptosis may not occur; however, monocyte chemoattractant protein-1 is still induced in the RPE, leading to the recruitment of macrophages to the choroid. The activity of these activated macrophages promotes the release of additional cytokines, which in turn contribute to further ROS production. These findings suggest that ROS-induced inflammation may play a role in amplifying oxidative stress by increasing ROS levels [68,69,84].

**Figure 3:**
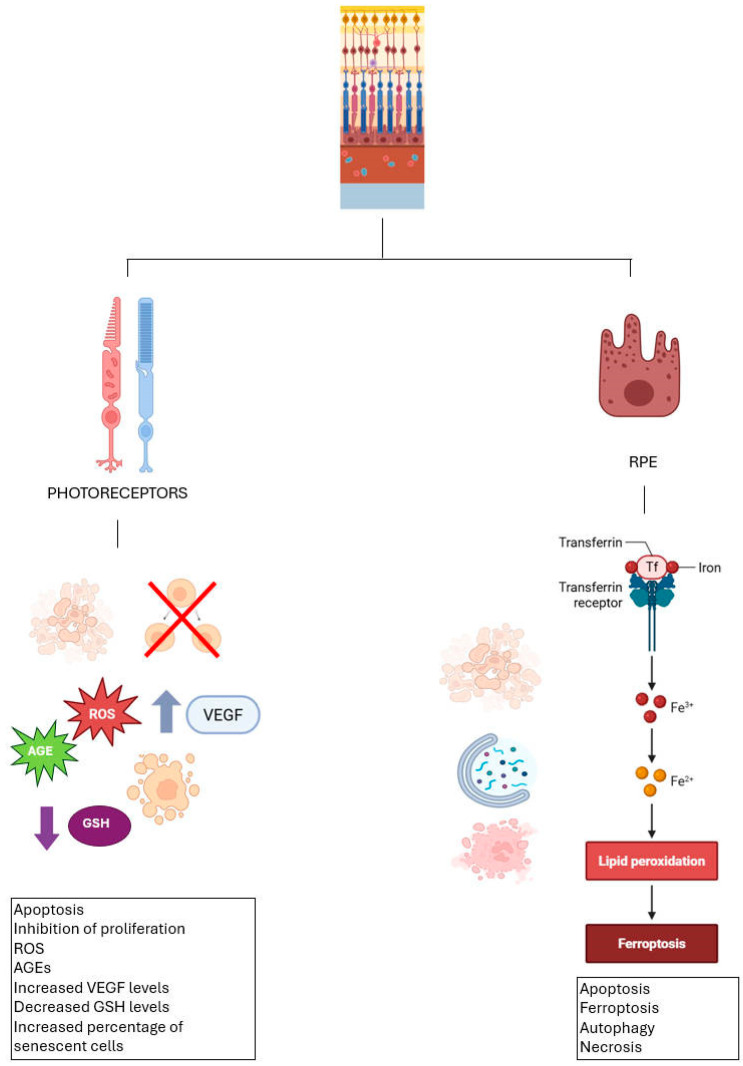
Prooxidant mechanism in retinal degeneration. The upward arrow indicates an increase in the levels, while the downward arrow indicates a decrease.

In the context of oxidative stress, secondary antioxidants are defined as enzymes that do not have a direct role in the elimination of ROS but have a preventive and supportive role. Secondary antioxidants include selenoproteins. Like other selenoproteins, GPX also follows a hierarchical priority in selenium utilization, with GPX1 and GPX4 being the most favored isoforms [32]. Six GPX variants are found in the ocular surface, with GPX1 and GPX4 predominating, while GPX3 is mainly localized in the sclera. A comparison of the expression levels of GPX and catalase (CAT) in different ocular districts suggests that the detoxification of H_2_O_2_ in the cornea and sclera is mainly carried out by GPX. However, under conditions of high H_2_O_2_ concentration, CAT may assume a more prominent role than the GSH-based redox system [60,61]. High levels of GPX, specifically GPX1, GPX3, and GPX4, are found in the lens. Given the lens’s high protein content, tight redox control over thiol groups is essential to prevent protein aggregation [85]. At the retinal level, superoxide dismutase (SOD), GPX, CAT are the most important in the defense against ROS. Specifically, in the presence of prolonged exposure to light, SOD and GPX become particularly representative. It is noteworthy that the retinal distribution of GPX isoforms differs from that of the cornea and lens, with GPX3 being the most abundant in the retina, followed by GPX4. Moreover, GPX3 represents an antioxidant enzyme with the highest expression levels in this ocular region. GPX3 localizes in the extracellular space, where it plays a protective role against the cell surface and basement membranes, contributing to the defense against oxidative stress in these structures [37]. Importantly, GPX1 shows high expression in the retina during the early stages of ocular development, suggesting a possible role as an antioxidant defense mechanism in the early stages of retinal maturation [34] (Figure 3).

Based on these data, it is not surprising that oxidative stress plays a key role in the development of various retinal diseases.

## 4. Oxidative Stress and Retinal Degeneration: A Link Worth Exploring

### 4.1. Age-Related Macular Degeneration

Advanced age is the main risk factor for age-related macular degeneration (AMD), a chronic eye disease that tends to occur after the age of 50. However, in industrialized countries, early cases are also observed in younger individuals. In the United States, AMD is one of the leading causes of blindness, with a distribution that varies among ethnic groups: it affects 54% of Caucasian patients, 14.3% of Hispanics, and 4.4% of African Americans. Currently, among white people over the age of 40, this condition is the leading cause of visual impairment and blindness [86]. With increasing longevity, an increase in the incidence of the disease is expected [87].

AMD can manifest itself in a dry or exudative form: while in the first case, the disease progresses more slowly, the acute form progresses rapidly. In some cases, the two forms may occur at the same time [88,89]. Comparing cones and rods, the former not only require more ATP than rods but are also more sensitive to oxidative stress [90].

Several endogenous and exogenous factors are known to influence the onset and course of the disease [91]. Among these, it is interesting to explore the role of ROS. In AMD, an increase in the production of advanced glycation products (AGEs) is observed because of molecular alterations due to ROS [89,92]. AGEs appear to modulate gene expression, promoting the aging of RPE cells and thereby contributing to disease progression [93,94]. A serious consequence is DNA damage, which becomes even more significant considering that photoreceptors and RPE cells lack mechanisms to detect such damage [88,89]. Furthermore, focusing on the RPE, the photoreceptors most commonly present are cones, which not only require more ATP than rods but are also more sensitive to oxidative stress [90].

In vitro and in vivo studies have found increased autophagic activity in the early stages of AMD, whereas this activity is attenuated in the advanced stages of the disease. It was found that reduced efficiency of the autophagic process makes RPE cells more vulnerable to oxidative stress. In contrast, enhanced activation of the autophagic system appears to offer a protective mechanism against oxidative damage. In response to oxidative stress, the organism activates a two-step defense mechanism. Firstly, enzymes such as the monooxygenase system of cytochrome P450, aldoketo reductase (AKR), carboxylesterases (CES), and epoxide hydrolase come into play, which promote oxidation and reduction reactions aimed at neutralizing toxic compounds. The second phase involves the conjugation of metabolites with hydrophilic molecules to facilitate their elimination. Direct and indirect antioxidants act in this phase. Direct antioxidants include SOD, GSH and Trx, which directly neutralize free radicals and rapidly regenerate. Indirect enzymes are involved in the synthesis and restoration of GSH and Trx levels and contribute to the removal of oxidized compounds [95].

An effective antioxidant defense cannot be achieved without the action of NRF2. Phase II antioxidants respond to the NRF2–KEAP1 (Kelch-like ECH-associated protein 1) transcriptional complex, which is a key element in the cellular response to oxidative stress. NRF2 plays a central role in maintaining redox homeostasis by activating transcription of antioxidant genes through binding to the Antioxidant Response Element (ARE) located in gene promoters. This factor regulates the expression of direct and indirect antioxidant enzymes [96,97,98].

The results obtained from studies on the concentration of GSH in the serum of patients with AMD are varied. Some studies have found no significant differences in GSH levels between subjects with AMD and healthy controls [99]. However, most studies have reported a reduction in serum GSH levels in patients with AMD compared to subjects without the disease [100,101,102]. In contrast to these data, a single study showed higher GSH levels in the AMD group than in controls [103]. This variability in results suggests that GSH alone is not a specific and reliable indicator for assessing the development or progression of AMD. Several studies have shown that under oxidative stress conditions a significant decrease in the ratio between GSH and GSSG is observed in RPE cells [34,85,95]. Only in rare cases have increased levels of intracellular GSH been observed [71,74]. More recent research is focusing on the analysis of mitochondrial glutathione (mGSH), as this compartment can contain 5 to 10-fold higher concentrations of GSH than the cytosol. GSH is transported within mitochondria via specific active transport systems and is involved in several essential functions: antioxidant protection, detoxification of xenobiotics, stabilization of mitochondrial DNA [104,105], and participation in the synthesis of iron-sulfur clusters, and redox regulation of the electron transport chain. Studies in RPE cells have shown that during the development of AMD, mGSH levels are significantly reduced [86,88,89]. The enzyme isoforms GPX1 and GPX4 appear to have a defensive function against the progression of AMD [35,36]. Research in Polish AMD patients has identified that mutations in the GPX1 gene are associated with reduced antioxidant capacity, which may have contributed to the onset of the disease in this group [92]. In parallel, studies in mouse models have shown that GPX4 can inhibit the increase in VEGF-A factor, the levels of which rise during neovascularization [35]. Further research has shown that reduced GPX enzyme activity is associated with an increased risk of developing AMD [106], and in several studies, affected patients were found to have lower enzyme activity than healthy subjects [89,107]. However, more recent data have found, in some cases, higher GPX activity in patients with AMD than in controls, suggesting a complex and potentially compensatory picture in the regulation of antioxidant activity [108]. Most research has either shown a reduction in GR activity in patients or indicated that reduced enzyme activity may increase the risk of developing the disease [106,107,109]. Lower GR activity has been correlated with a lower cellular antioxidant capacity [107]. In the context of AMD, various mechanisms contribute to retinal cell death, including hypoxia, elevated intraocular pressure (IOP), oxidative and nitrative stress, glutamate toxicity, neurotrophic factor deficiency, and autoimmune responses. All these data show a role for GSH in AMD [110].

### 4.2. Retinitis Pigmentosa

Retinitis pigmentosa (RP) is a mitochondrial disease characterized by dysfunctional mitochondria, which leads to the degeneration of photoreceptor cells. This impairs the retina’s ability to respond to light stimuli and ultimately results in vision loss. Among inherited retinal diseases, RP is the most common form in both humans and animal models, and it belongs to the group of disorders known as NARP (neurogenic muscle weakness, ataxia, and RP) [111]. Retinitis pigmentosa (RP) is a genetically heterogeneous disease caused by mutations in over 271 genes and 36 loci associated with inherited retinal dystrophies [111,112]. Among these, mutations in the gene encoding rhodopsin represent the most common cause of autosomal dominant RP, responsible for 20–30% of all cases [113,114]. Most of these mutations lead to abnormal folding of rhodopsin, which becomes trapped in the endoplasmic reticulum [115]. This abnormal accumulation activates the unfolded protein response (UPR) that causes stress in the endoplasmic reticulum, resulting in photoreceptor death [116]. Sizova et al. [117] observed that, in a rat model with autosomal dominant RP, rod death occurs via a mitochondria-mediated apoptotic mechanism. In the rod cells, pro-apoptotic mitochondrial proteins such as BAX, cytochrome C, and apoptosis-inducing factor (AIF) move from the mitochondria to the cytosol, initiating mitochondrial dysfunction. The increased production of ROS by mitochondria, the upregulation of BAX, and the reduction in the Bcl-2 protein led to the death of rods, followed by the death of cones. This mechanism has been confirmed in several animal models of RP [118,119].

Although the RP-related genes are mostly expressed in photoreceptors or RPE, many regulate mitochondrial functions. Three key cofactors of mitochondrial metabolism are effective in response to oxidative stress: alpha-lipoic acid (ALA), coenzyme Q10 (CoQ10), and carnitine (CARN). These compounds, also known as “mitochondrial nutrients” (MN), have been proposed as potential treatments not only for mitochondrial disorders but also for other diseases characterized by oxidative stress, in the context of so-called “mitochondrial medicine” [120,121,122]. The crucial role of oxidative stress in RP is demonstrated by the fact that several in vivo studies have found that administration of antioxidant compounds in mouse models of RP prevented cell death and thus loss of retinal function. Specifically, ALA, SOD2, CAT, and various plant-derived compounds were administered in different experimental protocols [123,124,125,126,127].

Vision loss begins with the degeneration of rod cells, followed by the death of cone cells. This process triggers a vicious cycle: oxidative damage accelerates cone cell death due to a progressive metabolic imbalance [128], a reduction in supportive factors [129], and damage caused by the death of rod cells [130,131]. Cones are unable to counteract the increase in oxidative stress, which leads to their death. This process is attended by a decrease in GSH and an increase in malondialdehyde and nitric oxide [50,132].

Several antioxidant treatments have proven effective in slowing cone cell death in animal models [123,132,133,134]. Furthermore, increasing the activity of natural antioxidant enzymes, such as SOD1, SOD2, and GPX, reduced oxidative damage and improved cone survival in RP mice [124]. It is worth noting that both primary mitochondrial DNA (mtDNA) mutations and secondary mitochondrial dysfunction, which impair mitochondrial or cytosolic proteins, can generate oxidative stress and contribute to the development of RP [135]. Although not all RP mutations occur in the mitochondria, oxidative stress is involved in the disease, both as a cause and consequence, in photoreceptors, neurons, and glial cells [136]. Punzo et al., demonstrated that rod death released ROS and Reactive Nitrogen Species (RNS) into the outer retina, damaging cones. Oxidative stress in cones can alter membrane polarization and mitochondrial redox balance, contributing to the progressive loss of their function [130]. It was also observed that, in animal models of RP, overexpression of antioxidant enzymes such as SOD, CAT and GPX in photoreceptor mitochondria reduces rod degeneration, limits oxidative damage and improves cone survival [124,137].

### 4.3. Diabetic Retinopathy

Diabetic retinopathy (DR) is a complication of type 1 and type 2 diabetes mellitus that affects the small blood vessels of the retina and is a major cause of vision loss globally [138,139]. According to 2022 data, about 22% of people with diabetes (about 103 million) have signs of DR, and 6% (about 28 million) suffer from an advanced form of the disease that can seriously impair vision [140]. Over the next 25 years, it is estimated that the total number of affected patients may reach 160 million. Of these, 45 million are expected to develop a vision-threatening form of the disease, and about 28 million will suffer from macular edema [140].

One of the main effects of excess glucose in cells is increased oxidative stress, which activates and amplifies numerous pathological mechanisms; in particular, superoxide is the most representative ROS in diabetes [141]. It has been seen that in mouse models of diabetic retinopathy, there is higher superoxide production than in controls [142,143,144]. Many complications of diabetes share common cellular pathological mechanisms, which can be traced back to increased oxidative stress [141,145]. These mechanisms include activation of protein kinase C (PKC), AGE formation, overregulation of the receptor for AGEs (RAGE) and its ligands, as well as increased activity of the polyol pathway [146,147]. All these factors are known to contribute to the development of diabetic complications [148,149,150,151].

From a molecular point of view, excessive superoxide production damages DNA and activates the enzyme poly (ADP-ribose) polymerase (PARP) [152]. This enzyme chemically modifies GAPDH by reducing its activity. Oxidative stress can also directly inactivate GAPDH by oxidizing a specific cysteine residue via hydrogen peroxide [153]. When glycolysis is interrupted at this level, its intermediates accumulate, which in turn feed the already active pathological processes. Oxidative stress, through inhibition of the enzyme GAPDH, alters the availability of glucose-derived metabolites and promotes the accumulation of highly reactive dicarbonyl compounds, leading to excessive production of AGE [152]. AGEs contribute to disease development by several mechanisms, for example, the impairment of the structure and function of the extracellular matrix. It has been observed that failure to form AGEs prevents thickening of the basement membrane in retinal capillaries of rats with diabetes [154]. The interaction of AGEs with specific receptors, such as RAGE, is also a particularly observed mechanism. Activation of RAGE can trigger several intracellular signaling pathways, including JAK-STAT (Janus kinase-signal transducer and activator of transcription), MAPK (mitogen-activated protein kinases), PI3K/Akt (phosphatidylinositol-3-kinase/Akt), and the Ras/Rac/Cdc42 cascades [155]. In addition, AGE generation is increased by the presence of ROS and, in turn, can further stimulate ROS production through the activation of Nox enzymes as well [156,157]. It has long been known that limiting AGE formation can slow the progression of DR in several animal models [148]. With diabetes, an increase in RAGE receptor expression is observed in rat retinal Müller cells; similarly, exposure to high glucose concentrations stimulated RAGE expression in the human Müller cell line MIO-M1 [158]. This deregulation appears to be mediated by the production of superoxide anion [159]. Suppression of AGE formation reduces Müller cell activation [160]. In MIO-M1 cells, RAGE activation resulted in the induction of proinflammatory mediators such as IL-6, IL-8, CCL2, and VEGF. Pericytes show decreased proliferative capacity and increased apoptosis when growing on AGE-modified basement membrane substrates [161]. However, treatment of cells with SOD or Nox inhibition counteracted oxidative stress and reduced pericyte apoptosis [162,163]. Advanced lipoxidation end-products (ALE) also play an important role in retinal pathological processes. In particular, Nε-(3-formyl-3,4-dehydropiperidino)lysine (FDP-lysine) accumulates in the Müller cells of diabetic rats [164]. This compound is capable of inducing apoptosis in MIO-M1 cells, as well as stimulating VEGF, IL-6, and TNF-α expression. Aldose reductase is an enzyme that catalyzes the conversion of glucose to sorbitol, a process that requires NADPH as a cofactor. It has been hypothesized that excessive consumption of NADPH by this enzyme contributes to oxidative stress [165]. Furthermore, fructose, produced later in the polyol pathway, is a more reactive substrate than glucose for the generation of AGEs [166]. Inhibition of aldose reductase is effective in limiting apoptosis of cultured retinal pericytes and preventing the onset of DR in rats, indicating that this metabolic pathway plays a crucial role in the pathogenesis [167,168,169,170]. An additional metabolite that accumulates under hyperglycemic conditions is DAG, known to be a potent activator of the β isoform of protein kinase C (PKC-β) [171]. Activation of PKC-β involves numerous effects at the cellular level, and its inhibition has been shown to prevent diabetes-related vascular changes in animal models [172]. In the early stages of DR, one of the pathological manifestations is impaired retinal blood flow. This phenomenon is attributable in part to PKC-mediated suppression of the expression of endothelial nitric oxide synthase (eNOS). The consequence of this process is a reduced production of nitric oxide by endothelial cells [173]. Blocking PKC-β restores retinal blood flow and reduces leucocyte adhesion to the vascular wall [80,174]. PKC-β also contributes to blood-retinal barrier dysfunction by facilitating occludin phosphorylation, a crucial step in VEGF-induced barrier breakdown [175,176]. Furthermore, hyperglycemia activates the transcription factor NF-kB, in a process dependent on PKC-β [177,178]. NF-κB is activated by oxidative stress and is a key regulator of inflammatory responses [179]. Its activation leads to the expression of proinflammatory cytokines such as IL-1β and TNF-α [180]. IL-1β, in turn, promotes the production of superoxide anion, activation of MAPK and proinflammatory caspases, and further activation of NF-κB [87]. NF-κB can induce the expression of genes for Nox subunits [181]. Taken together, this evidence suggests that PKC-β and NF-κB act as central players in proinflammatory signal transduction in DR. Alternative relevant mechanisms of cell death in the pathogenesis of DR have been proposed. Recent evidence suggests that ferroptosis may contribute significantly to retinal degeneration in this pathological context. Pharmacological inhibition of fatty acid binding protein 4 (FABP4) has proven effective in reducing lipid peroxidation, ROS accumulation, and iron levels in the retinas of diabetic mice, while preventing histological changes [182]. These protective effects appear to be mediated, at least in part, by activation of the nuclear receptor PPAR-γ, as demonstrated in ARPE-19 cells exposed to high glucose levels. Another damage mechanism involves the interaction between nitric oxide (NO) and superoxide (O_2−_), which leads to the formation of peroxynitrite, a highly toxic reactive species for lipids, proteins, and DNA. The genetic deletion of inducible nitric oxide synthase (iNOS) confers protection against several diabetes-induced retinal changes in mice, including superoxide overproduction, capillary degeneration, pericyte disruption, and leukostasis [183]. Although this study did not delve into the mechanism by which iNOS promotes the production of superoxide, it is plausible that this occurs via impairment of mitochondrial electron transport chain complexes caused by reactive nitric oxide intermediates.

In the diabetic retina, GSH levels decrease, while oxidized glutathione increases, as reported by several studies. In diabetes, enzymes responsible for glutathione regeneration are compromised, including GPX, GR and CGL which, as described above, play a crucial role. It is worth noting that GCL depends on the activity of the transcription factor NRF2, which is sensitive to the redox state of the cell. NRF2, under normal conditions, binds to specific regions of DNA, such as antioxidant response element 4 (ARE4), inducing the expression of the catalytic subunit of GCL. In diabetic retinopathy, however, a reduced transcriptional activity of NRF2 and a lower expression of GCL are observed, further contributing to the increase in oxidative stress in the retina [184].

Moreover, several studies have shown that the selenoproteome system, in particular GPX1 and GPX4, plays a fundamental defensive role against oxidative stress in the diabetic retina. These observations are supported by the fact that it is well documented that selenium treatment in DR model enhances the activity of these enzymes, counteracting pathological processes such as lipid peroxidation, cellular oxidation, and inflammation [185,186,187].

## 5. Counteracting Oxidative Stress: A Strategy for Treating Retinal Diseases

Considering the reported data, it is evident that ROS plays a crucial role in retinal diseases. A promising therapeutic strategy is to fight ROS by modulating antioxidant systems.

### 5.1. Small Antioxidants

Clinical studies have investigated the supplementation of small antioxidants (vitamins A and E, docosahexaenoic acid, beta-carotene, and lutein) as a therapeutic option for RP. Some of these compounds have shown a protective effect, reducing visual field loss and slowing the decline in retinal function [188]. However, a clinical study on patients with X-linked RP showed that long-term docosahexaenoic acid supplementation is not effective, although the number of participants was limited [189]. In rd1 mouse models, a combination of lutein, zeaxanthin, lipoic acid, and reduced GSH showed effective protection of photoreceptors, whereas the same antioxidants administered individually had no effect [190]. The possible therapeutic effect of small antioxidants has also been investigated in patients with AMD. The well-known AREDS study, started in 1992 involving 4757 participants, is a long-term prospective study aimed at evaluating the impact of oral antioxidant supplements on the progression of advanced AMD. The vitamin mixture used included vitamin C, vitamin E, β-carotene, zinc oxide, and cupric oxide. After five years of monitoring, the results showed a significant slowdown in the development of advanced forms of the disease [191,192].

### 5.2. KEAP1–NRF2 Pathway

Given the role of the KEAP1–NRF2 pathway in modulating the antioxidant response, it is not surprising that NRF2 is implicated in several ocular diseases [193]. In mouse models lacking NRF2, typical signs of AMD were found [194]. Although not unique, most studies show a functional reduction in NRF2 activity in DR. Recent studies have identified an alternative mechanism of NRF2 degradation mediated by the REDD1 (regulated in development and DNA damage response 1) protein, which promotes its phosphorylation by GSK-3β and thus its degradation. It is now clear that NRF2 plays a crucial protective role in DR. In mouse models, genetic deletion of NRF2 worsens the disease, leading to increased TNF-α expression, early deterioration of the blood-retinal barrier, and aggravation of visual dysfunction. In contrast, pharmacological activation of NRF2 has shown significant protective neurovascular effects in animal models, making this signaling pathway a promising therapeutic strategy for the prevention and treatment of DR [195,196]. In Pde6b-KO mice, models lacking phosphodiesterase 6B protein, increased expression of NRF2 resulted in the protection of the RPE and a decrease in cone damage. At the same time, the transcriptome analysis revealed that NRF2 activates several cellular pathways linked to protection from oxidative stress, particularly the biosynthesis of GSH [197]. Sulforaphane, an isothiocyanate of plant origin, has shown its therapeutic properties in experimental models of retinal degeneration by activating NRF2, resulting in increased levels of TXN, TXNRD, and heme oxygenase-1 (HO-1) [42,198]. Several new synthetic compounds with greater efficacy in activating NRF2 were evaluated in different models of ocular diseases. Among these, triterpenoid RS9, administered into the vitreous body for two weeks in a rabbit model of RP, resulted in a significant enhancement of NRF2 transcriptional activity and counteracted the ONL thickness reduction [199]. In most cases, NRF2 activators react with KEAP1 cysteines, forming covalent bonds through Michael reactions. However, at high concentrations, these compounds can also modify other cysteine-containing proteins, causing undesirable effects. An alternative and more targeted approach is to interfere with the interaction between KEAP1 and NRF2, to activate NRF2 more selectively and safely [200].

### 5.3. Gene Therapy

Another interesting therapeutic approach is based on gene therapy. Approaches based on the administration of TXN family proteins have been explored. Human RPE cells modified with an AAV vector containing the TXN2 gene showed increased resistance to lipid peroxide stress and apoptotic stimuli. Furthermore, TXN2 expression attenuated endoplasmic reticulum (ER) stress by increasing the production of heat shock proteins (HSP) [201]. Miranda and colleagues tested the efficacy of TXN by intraperitoneal injection in rd1 mice and observed protective effects: the treatment counteracted photoreceptor degeneration and reduced inflammation. An increase in GSH, useful for maintaining redox balance, was also observed [202]. Interestingly, the E. coli-derived form of TXN was used, which contains only the active site cysteine pair, thereby excluding any effects related to structural cysteine. Also, increasing the expression of proteins downstream of the TXN pathway, such as MSR and PRDX, showed promising results as a therapeutic strategy for retinal degeneration [203,204,205]. As discussed earlier in the development of RP, there is secondary degeneration of cones. This phenomenon has been elucidated with the discovery of rod-derived cone survival factor (RdCVF), a trophic protein that promotes cone survival [129]. RdCVF is encoded by the NXNL1 gene, specifically expressed in photoreceptors, and is secreted by rods. It binds to basigin-1 (BSG1), which is associated with the glucose transporter GLUT1 on the cone membrane, thus promoting glucose uptake and stimulating aerobic glycolysis [206]. The NXNL1 gene produces, via alternative splicing, a second protein called RdCVFL, which has a thioredoxin-like structure with a cysteine-based catalytic active site. An increased vulnerability to photo-oxidative damage is observed in mice lacking NXNL1. Viral administration of both proteins, RdCVF and RdCVFL, protected photoreceptors from degeneration caused by genetic mutations and exposure to light [207].

Cepko et al. demonstrated that systemic administration of insulin prolongs cone survival in a mouse model of RP by stimulating the insulin/mTOR pathway. Based on this idea, 20 genes related to glucose metabolism were tested in rd1 mice, but only TXNIP showed positive effects [208]. TXNIP binds to reduced TXN via a disulfide bond between cysteine 32 of TXN and cysteine 247 of TXNIP, inhibiting it. In addition, TXN IP inhibits glucose uptake by binding to GLUT transporters and promotes the utilization of alternative energy sources to glucose, such as lactate [209].

Administration of AAV containing TXNIP improved cone survival and visual function in rd1 mice. A mutant of TXNIP (C247S), unable to bind to TXN, showed an even stronger effect in protecting cones, suggesting that the interaction with TXN represents a negative control mechanism of TXNIP. Furthermore, co-administration of the enzyme lactate dehydrogenase B (LDHB), which converts lactate into pyruvate, further enhanced the benefits [210].

### 5.4. N-Acetyl-L-Cysteine

Research published in 2019 examined the antioxidant activity of NAC using it on primary RPE cell cultures obtained from individuals with AMD and healthy subjects. By analyzing ROS generation, cell viability, GSH, and ATP levels, it was found that prior administration of NAC decreased ROS production and provided protection against apoptosis. The treatment resulted in an improvement in mitochondrial function in both cell groups analyzed. Particularly remarkable is the fact that, only in cells derived from AMD patients, NAC significantly reduced ROS levels and increased GSH concentration. Furthermore, NAC’s protective ability against H_2_O_2_ induced GSH depletion and mitochondrial impairment was more pronounced in AMD cells than in healthy cells. These results highlight the therapeutic potential of NAC in counteracting oxidative stress at the RPE level. The positive data obtained in RPE cells from AMD patients reinforces the prospect of using NAC as a possible treatment for this degenerative disease [211].

### 5.5. Selenium

Selenium (Se) plays an essential role in human health, particularly in its organic variants such as selenomethionine (Se-Met), selenocysteine (Sec), and methyl-selenocysteine [212]. It represents the active compound and acts as a reserve for the production of selenocysteine, which constitutes the main metabolic pathway for the formation of selenoproteins [29]. Consequently, an insufficient dietary intake of selenium may impair selenoprotein synthesis. Supplementation with selenium may enhance the expression and activity of selenoproteins and related enzymes, thereby allowing the best possible efficiency [213]. A 2012 study investigated the efficacy of different selenium products in protecting the corneal epithelium. Initially, the effects of sodium selenite, SeMet, seleno-cysteine, selenium-containing peptides, and Se-lactoferrin on CEPI-17-CL4 cells and subsequently on an in vivo model of dry eye were evaluated. Not all selenium-containing molecules proved effective, but treatment with Se-lactoferrin eye drops at a concentration of 18 μM resulted in a significant reduction in the generation of 8-OHdG. This treatment alleviated corneal irritation and promoted the improvement of dry eye-induced lesions. Levels of oxidative stress markers at the corneal level also decreased significantly following the treatment. Several studies have investigated the therapeutic potential of eye drops containing selenium products to counteract oxidative stress. For example, Higuchi and colleagues also investigated the efficacy of a SeP-based eye drop and found an increase in GPX activity, while Ou and colleagues tested eye drops with copper selenide nanoparticles and documented the activation of the NRF2 pathway and an improvement in SOD2 and GPX1 activity [214,215]. Given these data, selenium-enriched ophthalmic preparations could be interesting therapeutic options for the treatment of ocular diseases related to oxidative stress [216].

Concerning DR, one study showed that a higher dietary intake of Se is associated with a protective effect [217]. Another study showed that Se can counteract the oxidative stress of hyperglycemia in an RPE cell line by protecting GPX activity [185]. Se nanoparticles (SeNP) appear to have a protective action against hypoxia damage in AR-PE-19 cells by reducing cell apoptosis. This effect could be due to the inhibition of a TRPM2 channel, resulting in decreased mitochondrial ROS, inflammation, and Ca^2+^ entry [218]. In addition, the effect of SeMet was tested on murine ARPE-19 and RPE; notably, pretreatment with SeMet one hour before exposure to H_2_O_2_ increased intracellular GSH levels, due to the activation of the SLC7A11 transporter [186,219].

### 5.6. Endogenous Redox Defenses

In the diabetic rat retina, there is a reduction in mitochondrial SOD activity (SOD2) [220]. Conversely, overexpression of SOD2 in transgenic mice confers significant protection against retinal vascular changes, such as the formation of acellular capillaries, typical of DR [143]. This overexpression not only counteracts the increase in superoxide production induced by hyperglycemia but also prevents the increase in mitochondrial membrane permeability and preserves the activity of complex III of the electron transport chain [221]. This evidence suggests that restoring or enhancing SOD2 activity may represent an effective therapeutic strategy to mitigate mitochondrial and vascular damage in the diabetic retina. Decoupling the mitochondrial proton gradient from ATP synthesis represents an endogenous mechanism that may reduce ROS production by mitochondria. Uncoupling proteins (UCPs), in particular UCP1–3, are activated by superoxide as a compensatory response to limit its further generation [222]. In cultures of bovine retinal endothelial cells and pericytes, RNA expression of UCP1 and UCP2 is increased in the presence of moderately high glucose concentrations, although this adaptive response is lost when glucose levels become excessive [223]. This has led to the hypothesis that mitochondrial uncoupling may initially activate as a protective mechanism but is then overwhelmed under conditions of severe oxidative stress. This hypothesis needs further experimental confirmation. In the diabetic rat model, retinal expression of UCP2 was reduced, and treatment with losartan, a type 1 angiotensin II receptor antagonist, was able to restore its levels [224]. Significant depletion of GSH reserves is observed in experimental models of diabetes: reduced levels of GSH were found in the retinas of diabetic rats, in the retinal mitochondria of diabetic mice, and in pericyte cultures in vitro treated with high concentrations of hyperglycemia [143,167,225]. It has been shown that the expression of GCLC, the catalytic subunit of glutamate-cysteine ligase, is suppressed in the diabetic rat retina due to epigenetic modifications of its promoter [226]. These data suggest that, in DR, the GSH apparatus is unable to survive with the increased production of ROS, thus impairing one of the cell’s key antioxidant defenses. Under physiological conditions, NRF2 is confined to the cytoplasm by association with KEAP1, which causes its polyubiquitination and subsequent proteasomal degradation. Under conditions of oxidative stress, the interaction between KEAP1 and NRF2 is lost, and therefore NRF2 drifts into the nucleus where it goes on to regulate ARE sequences. These sequences activate several genes (GCLC, HO-1, GSR, and SOD1) that play a key role in restoring redox balance [227,228,229]. In the rat, NRF2 is highly expressed in the retina, with higher levels than those found in the brain and liver [230]. In human and murine retinas, NRF2 is present in several cell types, but in particular is highly abundant in Müller cells, which play a key role in maintaining retinal homeostasis [231]. The multifunctional deacetylase SIRT1 plays a significant role in protecting against DR [232,233]. SIRT1 exerts its beneficial effects through deacetylation of transcription factors such as NF-κB p65 and FOXO1 (Forkhead box O1), resulting in a reduction in proinflammatory and prooxidative activity. In addition, SIRT1 suppresses the expression of p66Shc, a protein involved in the generation of ROS, by attenuating oxidative stress through the inhibition of Rac1 and Nox2 activation pathways [234]. Increasing evidence indicates that the expression of SIRT1 is reduced in the retina of diabetic mice, and that its overexpression can confer significant protection. The regulation of SIRT1 in the retina under diabetic conditions is also influenced by regulatory RNAs. It is noteworthy that the protective effect of SIRT1 is not limited to prevention: due to its epigenetic mode of action, the restoration of its activity could reverse already established damage, making it a promising therapeutic strategy not only prophylactic but also regenerative in the context of DR [235] (Table 2).

### 5.7. Advantages and Current Limitations

The therapeutic strategies described show an interesting approach in containing oxidative stress. Treatment with small antioxidants is easy to administer and has a good safety profile [188,191,192]. The activation of the KEAP–NRF2 pathway is characterized by a more targeted approach to sustain endogenous responses to oxidative stress and has provided promising results in in vivo models [193,194,195,196]. Gene therapy, on the other hand, offers the opportunity for lasting results by correcting molecular alterations in a targeted manner [202,203,205,208,209]. Therapies based on selenium, NAC, and, in general, aimed at strengthening endogenous defenses are effective in restoring redox balance and counteracting, as far as possible, cell death [211,213,216,222,225,226,231]. However, each of the approaches described has limitations. Antioxidants show variable efficacy among patients, and not all clinical trials confirm positive outcomes. NRF2 activators may have side effects related to poor selectivity, while gene therapy is characterized by limitations related to the safety of the vectors used and the precision of action on the target. As for therapies based on selenium implementation, further studies are needed to confirm their efficacy in patients [214,215,236].

We believe that in the future, for the development of more effective therapies, it could be useful to test combined approaches between these therapies and work by increasing the selectivity of the compounds while ensuring fewer side effects related to the specificity of the targets. In addition, it could be interesting to exploit personalized medicine to clarify markers of pathologies for each patient.

## 6. Discussion

The retina is particularly vulnerable to oxidative stress due to its high oxygen consumption, intense metabolic activity, and continuous exposure to light. The role of oxidative stress in contributing to retinal degeneration is well described and documented in the literature [237,238]. Throughout this review, we have examined how cysteine and selenocysteine contribute to the maintenance of redox balance and cellular protection in retinal tissue [1,2,3,4,5,6]. Cysteine is the precursor for GSH synthesis and determines its availability [13,14]. Its biosynthesis is finely regulated by endogenous and exogenous signals and occurs through the transsulfuration pathway [7,8,9,10,11,12]. Collected data suggest that insufficient cysteine availability results in a significant depletion of antioxidant activity, leading to damage at the level of the RPE [6,80]. Selenocysteine also plays a key role by enabling the catalytic activity of selenoproteins, which are essential for counteracting ROS [27,29]. Among these, GPXs, particularly GPX4, play a crucial role in protecting photoreceptors, as demonstrated by several murine models [38,39].

Cysteine and selenocysteine regulate cellular signaling, gene activation, and the preservation of protein function through post-translational modifications such as S-nitrosylation [16,43,52,53,54,55]. For example, TXNs inhibit the pro-apoptotic activity of ASK1 by forming a disulfide bond with the C250 residue, and the absence of this complex correlates with apoptotic events in the retina [51,52].

## 7. Conclusions

Alterations in the availability or metabolism of these amino acids have been linked to the pathogenesis of major retinal diseases such as AMD, RP, and DR. In AMD, there is an accumulation of AGE, a reduction in GSH levels, and inhibition of the autophagic process, mechanisms that collectively contribute to the deterioration of the RPE. However, contradictory data regarding serum GSH levels in AMD patients suggest the need to analyze mGSH levels within this pathology. In RP, the loss of rod photoreceptors leads to secondary oxidative stress that damages cone cells. GSH deficiency, increased levels of NO and malondialdehyde, as well as mitochondrial dysfunction, contribute to degeneration. In vivo studies have demonstrated that overexpression of antioxidant enzymes, such as SOD2, CAT, and GPX, protects photoreceptors. Furthermore, mitoprotective molecules, including alpha-lipoic acid, coenzyme Q10, and carnitine, have been shown to efficiently decrease damage. In DR, oxidative stress is the central event that activates classical pathogenic pathways, leading to the formation of AGEs, activation of PKC-β, NF-κB-mediated inflammation, and mitochondrial damage. Within this context, mitochondrial DNA damage and reduced activity of enzymes such as SOD2 and GPX compromise the efficacy of endogenous antioxidant defenses.

The therapeutic strategies analyzed demonstrate significant potential: the use of NAC has shown positive effects in restoring GSH levels and reducing apoptosis in RPE cells derived from AMD patients. Similarly, the application of organic selenium compounds, including selenocysteine, SeMet, and SeNPs, has proven effective in enhancing antioxidant activity, reducing inflammation, and modulating the NRF2 response. Gene therapy-based interventions, such as the overexpression of TXN2 or viral delivery of RdCVF and RdCVFL, suggest that targeted activation of antioxidant pathways may provide both functional and structural protection to the retina. Additionally, modulation of the interaction between TXNIP and TXN has shown neuroprotective effects.

The Keap1-Nrf2 signaling pathway emerged as a central regulator of the antioxidant response. Activation of Nrf2 promotes the expression of genes involved in GSH synthesis and ROS detoxification, including GCLC, HO-1, and SOD. Several studies have demonstrated that pharmacological activation of Nrf2 or inhibition of its degradation can protect retinal cells from oxidative damage. Moreover, the thioredoxin system, including TXN and its regulators such as TXNIP, has shown therapeutic potential in models of RP, where it modulates redox signaling and supports cone survival.

Additional strategies, such as the use of mitochondrial nutrients (such as alpha-lipoic acid, CoQ10, and carnitine), selenium-enriched eye drops, and the modulation of ferroptosis-related pathways, have also shown promise in preclinical studies. These findings suggest that targeting cysteine- and selenocysteine-dependent antioxidant systems may offer effective therapeutic approaches for preventing or counteracting the progression of retinal degenerative disorders. However, while preclinical studies are promising, further research is needed to translate these findings into effective clinical therapies. A deeper understanding of the redox landscape in retinal cells will be essential for developing targeted approaches to ameliorate the progression of vision-threatening conditions.

In summary, this review demonstrates that cysteine and selenocysteine act as central pathophysiological regulators in the retina, both as precursors of antioxidants and as structural components of redox-sensitive enzymes. Their modulation represents a promising frontier for the prevention and treatment of degenerative retinal diseases. A deeper understanding of redox dynamics in the retinal context opens new diagnostic and therapeutic avenues. The modulation of cysteine and selenocysteine, as well as related enzymatic systems, represents a promising pharmacological strategy to counteract retinal neurodegeneration.

## 8. Future Perspectives

Considering the evidence presented, several promising research directions emerge that deserve further investigation. A deeper understanding of the cellular distribution of seleno-proteins across various retinal cell types, including RPE, photoreceptors, Müller cells, and endothelial cells, could be achieved using advanced single-cell transcriptomic and proteomic techniques [20,21,22,24,25,26,40]. Alongside this, elucidating the regulatory mechanisms of the enzymes CBS and CSE is crucial to better elucidate the strategy by which cells prioritize the synthesis of cysteine, GSH, hydrogen sulfide, or taurine under conditions of oxidative or inflammatory stress [10,11,12].

Further studies are needed to clinically validate antioxidant compounds such as NAC, SeMet, and GPX mimetics. These studies should ideally be conducted through randomized controlled trials, using both functional endpoints, for example, electroretinography and optical coherence tomography, and molecular readouts, such as redox biomarkers, NRF2 transcriptional activity, and intracellular GSH or GPX levels [66,209,210,212,213,214,239].

At the same time, the development of gene therapy strategies customized to retinal diseases represents an exciting possibility. Retina-specific adeno-associated virus vectors capable of inducing the overexpression of redox-related proteins such as TXN2, GPX4, or RdCVFL have already demonstrated efficacy in preclinical models of AMD and RP [186,220,221,222,223,224,225].

In parallel, the optimization of selenium-containing ophthalmic formulations is showing potential in reducing markers of corneal oxidative stress while activating protective antioxidant pathways [196,232,233]. Another promising strategy involves the development of molecules that can selectively interfere with the KEAP1–NRF2 complex. Unlike classical electrophilic activators, these targeted modulators may avoid off-target effects while promoting a more controlled activation of the antioxidant response [185,215,216,217,218,219].

Moreover, the interplay between metabolism and oxidative stress deserves further investigation. Modulation of key factors such as TXNIP, RdCVF, and lactate metabolism may offer a possible strategy to preserve cone function in the context of RP [229,230]. At the epigenetic level, the regulation of glutathione metabolism and redox signaling by elements such as p66Shc, GCLC, and SIRT1 appears to be a promising therapeutic target, particularly for restoring antioxidant capacity in diabetic patients [197,205,206,207,208].

Finally, the development of redox-based biomarkers for early diagnosis and therapeutic monitoring of retinal diseases represents a crucial step toward precision medicine. Candidate markers include mitochondrial GSH levels, NRF2 expression profiles, and the enzymatic activity of GPX4 or thioredoxin reductase in both ocular and systemic samples [105,200,202].

Taken together, these lines of research highlight the potential of an integrated, redox-centered approach that combines precise modulation of antioxidant pathways with innovative delivery platforms and molecular monitoring to define the future of personalized therapies in retinal degenerative disorders.

## Figures and Tables

**Table 1:** Main retinal selenoproteins, functions and localization.

Selenoprotein	Function	Distribution	Characteristic	Ref
GPX1	Reduces H_2_O_2_ and organic peroxides using GSH	High expression during early retinal development	Major defense against oxidative stress; supports early retinal protection	[30,34,35,36]
GPX3	Extracellular detoxification of peroxides	The most abundant GPX isoform in the retina; extracellular space	Protects cell surfaces and basement membranes	[37]
GPX4	Reduces lipid hydroperoxides and inhibits ferroptosis	Inner segments of photoreceptors, RPE, and choroid	Essential for photoreceptor survival; regulates lipid peroxidation	[35,38,39]
TXNRD	Regenerates reduced thioredoxin using NADPH	Retinal ganglion cell, inner nuclear layer, photoreceptors	Declines in mature retina are essential for redox signaling	[40,41,42]
MSRB	Reduces oxidized methionine residues in proteins	Ubiquitous; systemic antioxidant role	Less specific to the retina, but it contributes to redox balance	[27,31]
Selenoprotein P	Selenium transport in plasma: minor antioxidant function	Systemic; marginal retinal expression	Mainly involved in selenium homeostasis	[33]

**Table 2:** Therapeutic strategy to counteract oxidative stress in retinal diseases.

Therapeutic Strategy	Therapeutic Target	Methods	Treatment Outcomes	Models	Ref
Small Antioxidants	rod, cones, macula, photoreceptor outer segment membranes and retina.	Supplementation with vitamins A, E, beta-carotene, lutein, DHA	Slowing of visual decline in RP and AMD.	RP patients,10–50 years old, 29 men/33 woman; 4757 participants aged 55 to 80 with or without AMD.	[188,189,190,191,192]
KEAP1–NRF2 Pathway	BRB, retina, Müller cells, RPE, I/R retina, retinas of diabetic Long Evans rats.	Activation of NRF2 pathway	Protection against oxidative damage in AMD and DR; involves REDD1-mediated degradation, activation of HO-1, GSH biosynthesis, and TXN expression.	NRF2-deficient mice; DR mice, Sprague Dawley rats; I/R mice; Streptozotocin-Diabetic Rats.	[193,194,195,196,197,198,230,231]
Gene Therapy	rod, cones, vitreous, RPE cells, retinal ganglion cell.	AAV-mediated delivery of TXN2, MSR, PRDX, RdCVF/L genes	Improved retinal survival; modulates redox balance, ER stress, and glucose metabolism.	Neural retina of C57BL/6J mice; Pro347Leu rabbits; NRF2 KO mice; retinal epithelial cells; rd1 mouse; hypoxia-induced retinal ganglion cell; retinal pigmented epithelium cells; cone-enriched cultures from chicken embryos	[129,199,200,201,202,203,204,205,206,207,208]
N-acetyl-L-cysteine (NAC)	RPE, photoreceptors.	NAC treatment	Increase in GSH, improvement of mitochondrial function, reduction in ROS and protection of RPE cells, particularly in AMD patient-derived cultures.	Human retinal pigment epithelial cells	[211]
Selenium	oxidative metabolism, respiratory kinetics, cone, plasma, cornea, corneal epithelium, RPE.	Selenium compounds (e.g., Se-lactoferrin, SeNPs, SeMet) enhan	Enhancement of GPx and SOD2 activity; reduce oxidative stress, inflammation, and protection against dry eye and DR-related damage.	TKO mice; TXNIP^fl/fl^ mice; TXNIP^SKM−/−^ mice; RP mice; 98 healthy Chinese subjects; human retinal pigment epithelial cells; Sprague Dawley rats; human corneal epithelial cell; C57BL/6J mice; 415 Chinese diabetic patients with or without DR.	[29,185,209,210,212,213,214,215,216,217,218]
Endogenous Redox Defense	Retinal mitochondria, retinal pericytes, RPE, vascular and neuronal retina, retinal microvessels.	Enhancing SOD2, UCPs, GSH system, NRF2, and SIRT1 activity	Protection of retinal cells from hyperglycemia-induced oxidative and vascular damage, especially in DR.	Diabetic mice; retinal pericytes; human retinal pigment epithelial cells; bovine retinal endothelial cells; retinal capillary endothelial cells and pericytes; spontaneously hypertensive rats; normotensive Wistar–Kyoto rats; human eye globes; NRF2^−/−^ CD-1 mice; neonatal rat cardiac myocytes; mouse macrophage; mouse fibroblast; SIRT1-overexpressin mice	[143,167,186,219,220,221,222,223,224,225,226,227,228,229,232]

## Data Availability

No new data were created or analyzed in this study.
